# The Saudi Spine Society guidelines on spinal surgery during the COVID-19 pandemic

**DOI:** 10.1186/s13018-020-01732-4

**Published:** 2020-06-08

**Authors:** Ahmed Alturkistany, Fahad H. Abduljabbar, Fahad Alhelal, Nayef Bin Dajim, Salahaddeen Khalifah, Faisal Konbaz, Sami Aleissa, Amro Al-habib, Maan Kattan, Yahya Alqahtani, Raheef Alatassi

**Affiliations:** 1 Department of Surgery, Division of orthopedic surgery, King Faisal Specialist Hospital & Research Centre, Jeddah, Saudi Arabia; 2 Department of Orthopedics, Faculty of Medicine, King Abdulaziz University, Jeddah, Saudi Arabia; 3 Orthopedics division, Department of surgery, King Abdulaziz medical city, National Guard Health affairs, Riyadh, Saudi Arabia; 4 Spine Care Department, Neurosciences Centers, King Abdullah Medical City, Makkah, Saudi Arabia; 5 Division of Neurosurgery Department of Surgery, College of Medicine, King Saud University, Riyadh, Saudi Arabia; 6 Department of Anesthesia, King Abdulaziz medical city, King Saud bin Abdulaziz University for Health Sciences, Jeddah, Saudi Arabia; 7 Department of Surgery, Security Forces Hospital, Riyadh, Saudi Arabia; 8 Department of Orthopaedic Surgery, University of Western Ontario, London Health Sciences Center, London, ON Canada

In March 2020, the World Health Organization declared the novel coronavirus disease (COVID-19), which emerged in China at the end of 2019, a pandemic. By mid-April, severe acute respiratory syndrome coronavirus 2 (SARS-CoV-2), the virus that causes COVID-19, had infected more than a quarter of a million people globally [1–3]. Although the majority of those with infection experience mild illness, transmission can occur from asymptomatic individuals [3–5]. The unpredictability and rapid spread of the disease necessitate several, critical, and immediate measures to protect healthcare workers, patients, and the community.

National healthcare systems are focusing their resources to increase SARS-CoV-2 testing, to manage COVID-19 cases, and to implement preventive measures. However, during this pandemic, the need for urgent surgery will not stop. Both medical and surgical priorities have changed since the announcement of the pandemic, and they continue to evolve. Several hospitals in affected countries have taken immediate and unprecedented actions regarding patients awaiting surgery including postponing outpatient and elective surgery, canceling unnecessary operations, and suspending teaching sessions until further notice [5–8]. Due to this uncertainty and individuality in the decisions, it is incumbent on medical associations and societies to establish guidelines to organize workflow and protecting patients and healthcare workers.

As health care providers, we all have responsibilities towards patients, families, and our community. Therefore, the Saudi Spine Society (SSS) Scientific Committee developed a basic protocol with different levels of care to help hospital systems, especially departments dealing with spine patients, to provide optimal care and to help spine care practitioners and residents to cope with the situation.

## Surgical spine surgeries

The SSS recommends that spine surgery candidates be categorized into three categories: category A (immediate), category B (urgent), and category C (elective). These categories are defined as follows:

*Category A (immediate)* includes patients who need immediate surgical intervention within 24 h. To fall in this category, patients must have clinical or radiological evidence of neurological deficit or structural instability secondary to traumatic, infectious, degenerative, or oncological conditions.

*Category B (urgent)* includes patients who require urgent surgical intervention, generally within 72 h. Such patients will have imminent neurological deficit or structural instability due to traumatic, infectious, degenerative, or oncological conditions, which may lead to deterioration of their functional status. It also includes patients with infectious or malignant conditions of the spine even in the absence of neurological deficit or structural instability, who need close monitoring as they might develop a need for immediate surgical intervention.

*Category C (elective)* includes patients suffering from chronic or subacute spine disorders, other than oncological or infectious diseases, who may eventually need surgical intervention. For patients to fall in this category, they must not display any clinical or radiological evidence of imminent neurological or structural instability, which includes cases of spine trauma that could be treated conservatively.

For patients in categories A and B, surgical procedures should be carried out as usual, without any delay. Because these procedures are non-deferrable, and any delay may have a permanent negative effect on the functional outcome or may be life-threatening. Alternatively, other less invasive options may be considered depending on hospital resources and patient status. Each patient should be provided with a full explanation about symptoms of COVID-19 and its identification. Use of personal protective equipment is recommended by the Centers for Disease Control for every operative procedure performed on a patient with confirmed COVID-19 infection or a patient where there is suspicion for infection [9]. Finally, patients should sign the consent after knowing the risk of undergoing the surgery in this situation.

For patients in category C, we strongly recommend postponing the procedure unless the patient progresses to category A or B. Nevertheless, the risk of neurologic deterioration and quality of life must be considered, and we recommend that surgeons repeatedly assess the risk–benefit ratio of surgery. Patients must fully understand that the postponement of surgery is in their best interest, as well as that of the medical staff.

## Other spine interventions

All other elective surgical interventions including epidural steroid injections, nerve root blocks, and facet injections should be suspended and rescheduled unless the pain is intolerable.

## Spine physiotherapy and rehabilitation services

To reduce the number of people in the hospital, physiotherapy should be provided only to immediate postoperative patients and should be replaced by home exercise programs among other patients. Utilizing virtual platforms for providing different physiotherapy exercises is currently the best option.

## Spine outpatient services

Unnecessary visits to outpatient clinics should be minimized, and non-urgent visits should be canceled. Outpatient consultations can be replaced by virtual consultations using telemedicine. Spine care practitioners should contact each patient with a scheduled visit to evaluate whether they need to attend the clinic or not. Patients who need to attend the clinic must follow the social distancing measures. Additionally, spine care practitioners must follow their local institutional policies for outpatient visits during the COVID-19 pandemic.

## Fellowship, residency, and undergraduate spine training

All undergraduate clinical rounds and intrahospital teaching sessions must be canceled and switched to online platforms. All teaching activities for residents and fellows must also be switched to online platforms. The number of physicians in attendance during rounds, operations, on-call, and clinics should be minimized, using a team approach. Residents and fellows should be allowed to have alternative regular duties, with self-isolating themselves in their home for 2-week periods. All exams must be rescheduled. Academic affairs in each hospital should consider stopping residents from rotating to other hospitals for the next block “3 months”. Program directors must discuss ways to address deficiencies in training due to the COVID-19 pandemic.

## Spine conferences and workshops

SSS promptly postponed all their conferences, workshops, and events. We encourage other medical societies and associations to follow this and either defer or cancel such activities. We recognize that these events provide vital educational and networking opportunities; therefore, we recommend, that societies consider offering these events virtually through other communication platforms.

## Data Availability

Not applicable.

## Abbreviations

COVID-19: Coronavirus disease
SARS-CoV-2: Severe acute respiratory syndrome coronavirus 2
SSS: Saudi Spine Society
